# Intentionally ending one's own life in the presence or absence of a medical condition: A nationwide mortality follow-back study

**DOI:** 10.1016/j.ssmph.2021.100871

**Published:** 2021-07-15

**Authors:** Martijn Hagens, H. Roeline W. Pasman, Agnes van der Heide, Bregje D. Onwuteaka-Philipsen

**Affiliations:** a Amsterdam Public Health Research Institute (APH), Department of Public and Occupational Health, Amsterdam UMC, Location VU Medical Center, Office A-325, Van der Boechorststraat 7, 1081 BT, Amsterdam, the Netherlands; b Amsterdam Public Health Research Institute (APH), Department of Public and Occupational Health, Amsterdam UMC, Location VU Medical Center, Van der Boechorststraat 7, 1081 BT, Amsterdam, the Netherlands; c Department of Public Health, Erasmus MC, University Medical Center, Dr. Molewaterplein 40, 3015 GD, Rotterdam, the Netherlands

**Keywords:** The Netherlands, Suicide, Voluntarily Stopping Eating and Drinking, Self-ingesting self-collected medication, Euthanasia, Physician Assisted Dying

## Abstract

In the Netherlands, people who wish to intentionally end their own life can request for physician assistance in dying (PAD). Having a classifiable medical condition is a prerequisite to receive PAD. Some people, either in the presence or absence of a medical condition, choose to end life without assistance from a physician. This study estimates the frequency of people who intentionally ended their own life, and describes their demographic and medical characteristics through a nationwide mortality follow-back study based on questionnaires from certifying physicians of a stratified sample of death certificates of people drawn from the central death registry of Statistics Netherlands (n = 7277). In 1.85% of all deaths in 2015 people intentionally ended their own life; of which 0.50% by voluntarily stopping eating and drinking, 0.20% by self-ingesting self-collected medication, and 1.15% using other methods. Estimating the frequency of suicide is influenced by definitions and the information sources. The great majority of people who ended life by voluntarily stopping eating and drinking were over 80 years old and suffered from an accumulation of health problems related to old age, somatic problems, and/or dementia. People who ended their own life through other methods were mostly under 65 years old and primarily suffered from psychiatric, psychosocial and existential problems. Few people who intentionally ended their own life requested PAD, especially those who suffered from solely psychiatric diseases and those without a medical condition. PAD in the Netherlands is embedded in the medical domain as it is currently understood by Dutch law. This raises the question how to address the desire to die from people whose wish to intentionally end their own life is not rooted in a medical condition and therefore fall outside this medical framework of assistance in dying.

## Highlights

• In the Netherlands, in 1.85% of all deaths people intentionally ended their own life.
• Death by voluntarily stopping eating and drinking accounts for 0.50% of all deaths.
• The majority of people who intentionally ended their own life had medical conditions.
• Few people who intentionally ended their life requested physician assistance in dying.

## 1 Introduction

In the Netherlands, people who wish to end their own life have the possibility to request physician assistance in dying (PAD) under the Dutch Termination of life on request and assisted suicide review procedures Act (Termination of life and assisted suicide review procedures Act, 2001). To have this request granted, physicians need to meet the six criteria of due care that are laid out in the act. One of the key criteria being the physician has to “be satisfied that the patient's suffering is unbearable, with no prospect of improvement” (Termination of life and assisted suicide review procedures Act, 2001). The Dutch Supreme Court set the additional requirement that the suffering of the patient that motivates his decision to end his life should be caused by a medically classifiable condition. This emphasizes that PAD is embedded in the medical domain as it is presently understood in Dutch law (Dutch Justice Supreme Court, 2002). Nevertheless, about two thirds of physicians regard performing PAD as inconceivable for medical conditions like dementia or psychiatric diseases (Bolt, Snijdewind, Willems, van der Heide, & Onwuteaka-Philipsen, 2015). In 2015, almost half (45%) of all requests for PAD have not resulted in PAD (Onwuteaka-Philipsen et al., 2017); for requests of people with a psychiatric illness or with dementia the corresponding figures are 58% and 57% (Evenblij, Pasman, van der Heide, Hoekstra, & Onwuteaka-Philipsen, 2019).

People who wish to intentionally end their own life can also do this without assistance from a physician. This can occur in the presence of a medical condition, but also in the absence of a medical condition, for example when someone suffers from existential suffering (Van Wijngaarden et al., 2015). Some suicides involve violent methods like jumping from a great height, while others involve non-violent methods like self-ingesting self-collected medication (MED) or voluntarily stopping eating and drinking (VSED). These two non-violent methods to end life are specifically described in Dutch literature (Chabot, 2019; KNMG, 2011). Some people who choose to intentionally end their own life do so after their request for PAD was denied, while others believe PAD will not be an option for them or prefer to take their own responsibility for their death instead of burdening the physician (Hagens, Onwuteaka-Philipsen, & Pasman, 2017).

From 2014 to 2019, Statistics Netherlands estimated the number of suicides between 1.2 and 1.3% of all annual deaths (Statistics Netherlands, 2020a). Estimates for deaths by VSED range from 0.4% to 2.1% of all annual deaths, for deaths by MED from 0.2% to 1.1%, and for deaths by other or unknown methods at 1.0% (Van der Heideet et al., 2012; Chabot & Goedhart, 2009). Ending one's own life often happens in the context of a serious psychiatric and/or somatic medical condition (Van der Heide et al., 2012; Chabot & Goedhart, 2009; Statistics Netherlands, 2020b). However, it can also occur in the absence of a medical condition, for example, when older people are tired of living without having medical conditions. In this study, we aim to estimate the frequency of people ending life themselves and to obtain insight in the extent to which this in done in the context of a medical condition. We will answer the following research questions:

1. What is the frequency of VSED, MED, and other methods of ending one's life?
2. What are the demographic and medical characteristics of persons who end their lives through VSED, MED, or other methods?
3. What are the demographic and medical characteristics of persons who end their own life and have solely psychiatric diseases, other medical conditions or no medical conditions?

## 2 Method

### 2.1 Design and population

In 2015, a nationwide mortality follow-back study was performed, based on pre-structured questionnaires sent to attending physicians of a stratified sample of death certificates. This study was largely similar to previous mortality follow-back studies done in 1990, 1995, 2001, 2005 and 2010 (Van der Maas et al., 1996, Onwuteaka-Philipsen et al., 2003, Van der Heide et al., 2007, Onwuteaka-Philipsen et al., 2012, Van der Heide, Brinkman-Stoppelenburg, van Delden, & Onwuteaka-Philipsen, 2012, Van der Heide, van Delden, & Onwuteaka-Philipsen, 2017; Van der Maas et al., 1991). A stratified sample of death certificates of persons who died between August 1st and December 1st^,^ 2015 was obtained from the central death registry of Statistics Netherlands. All death certificates in that period were stratified based on the likelihood that the death had been preceded by an end-of-life decision. A special stratum was created when the reported cause of death was suicide. Cases that clearly precluded end-of life decision-making were retained in the sample, but no questionnaires were sent out to the physician (n = 384).

For the calculation of the frequency of VSED, MED and other methods of ending one's own life, the cases from the returned questionnaires (n = 7277) plus the cases that precluded end-of life decision-making (n = 384) were included (n = 7661). For the calculation of the demographical, medical, and care characteristics of persons who ended their life through VSED, MED or other methods we included all cases from the stratum containing suicides plus all cases where the physician had indications that the patient intentionally ended one's own life. Eight cases were excluded because answers by the physician to open ended questions clarified that suicide was not the cause of death. This resulted in a total number of 521 cases of people who ended their life. We distinguished between VSED (n = 25), MED (n = 73), other non-violent methods of ending one's life that left the body physically intact (e.g. helium, and plastic bag) (n = 44), violent methods (e.g. jumping from heights) (n = 256), and unknown methods (n = 123).

### 2.2 Questionnaire

Certifying physicians of the sampled cases received a written questionnaire containing twenty-nine questions focussing on end-of-life decisions that might have preceded the death of the patient involved. This questionnaire was largely similar to previous mortality follow-back studies (Van der Maas et al., 1996; Onwuteaka-Philipsen et al., 2003; Van der Heide et al., 2007; Onwuteaka-Philipsen et al., 2012; Van der Heideet et al., 2012; Van der Heide, van Delden, & Onwuteaka-Philipsen, 2017; Van der Maas et al., 1991). Amongst others, it contained questions about the underlying medical condition and whether the patient had requested PAD under the Dutch PAD law. Two questions focussed on patients intentionally ending their own life, namely ‘Do you have indications that the patient intentionally ended his or her life (without direct help of a physician)?’ and ‘which method was used: stopping eating and drinking (with or without care from a physician), self-ingesting self-collected medication, or other, namely … ?’. The data collection procedure precluded identification of physician and patient. The Ministry of Justice gave a guarantee that no physician could be prosecuted on the basis of information given to the researchers. A reminder was sent to those who had not returned the questionnaire. Of the 9351 questionnaires sent, 7277 were returned (response 78%).

### 2.3 Analysis

Statistical analyses were carried out using IBM SPSS version 25 (IBM Analytics). The results were made representative of all deaths during 2015 (n = 145,134) by weighting the data for stratification (sampling fractions ranged between 1/12th and 1) and missing numbers. Missing observations were not imputed as the numbers of missing observations were lower than 5%. Due to this procedure, the percentages reported cannot be derived from the absolute unweighted numbers. 95% confidence intervals were calculated. For the analysis of the context of a medical condition, data from people with a psychiatric disease were separately analysed as separate guideline exist to address requests for assistance in dying from this group of patients (Dutch Federation of Medical Specialist, 2018).

### 2.4 Ethics approval

According to Dutch policy the study did not require review by an ethics committee (Dutch Personal Data Protection Act, 2000).

## 3 Results

### 3.1 The incidence of VSED, MED, and other methods of ending one's own life

The total number of people who ended their own life in the Netherlands in 2015 is estimated at 1.85% of all deaths. Almost a quarter of this percentage (0.50% of all deaths) consisted of people passing away by VSED (see Table 1).

**Table 1:** Incidence of voluntarily stopping eating and drinking (VSED), self-ingesting self-collected medication (MED), and other methods of ending one's own life in 2015 in the Netherlands (n = 7661).

	n	Weighted rounded n (95% CI)	Weighted % of all deaths (95% CI)
VSED^a^	25	730 (530–980)	0.50 (0.36–0.67)
MED^b^	73	280 (150–430)	0.20 (0.11–0.31)
Other methods:	423	1690 (1370–2070)	1.15 (0.93–1.41)
- Other non-violent^c^	44	190 (100–340)	0.13 (0.07–0.23)
- Violent^d^	256	1030 (790–1340)	0.70 (0.54–0.91)
- Method not specified	123	470 (310–670)	0.33 (0.22–0.47)

a VSED = Voluntarily Stopping Eating and Drinking; none of the VSED cases had suicide registered as official cause of death.

b MED = self-ingesting self-collected medication; of the MED cases there were 3 cases (unweighted) in which suicide was not registered as cause of death (all other cases were registered as suicide).

c Non-violent methods include suffocation using helium or other gas and/or a plastic bag or intoxication.

d Violent methods include hanging, jumping in front of a train or from a high place, the use of a gun or a knife, drowning, and fire.

The remaining 1.35% were categorized into non-violent and violent methods of ending one's own life. Non -violent methods covered 0.33% of all deaths, consisting of MED (0.20%) and other methods like suffocation using helium, a plastic bag or intoxication (0.13%). Violent methods like hanging, jumping from a high place, drowning, or using a gun or a knife occurred in 0.70% of all deaths.

### 3.2 Demographic and medical characteristics of people intentionally ending life by VSED, MED or other methods

The majority of the people who intentionally ended life by VSED were female (76%), over 80 years (82%), and widowed (70%) (see Table 2). According to the physician, almost all (94%) had (a combination of) an accumulation of health problems related to old age (63%), somatic disease (61%) and/or dementia (34%). They had little psychosocial or existential problems (3%) and no psychiatric illnesses (0%). Almost half (45%) requested their physician for PAD under the Dutch PAD law.

**Table 2:** Characteristics of people who ended life by voluntarily stopping eating and drinking (VSED), self-ingesting self-collected medication (MED) and other methods of ending one's own life (n = 521; absolute unweighted numbers and weighted percentages)^a^.

	VSED^b^		MED^c^		Other methods of ending one's own life					
					Other non-violent		Violent		Method unknown	
	N = 25		N = 73		N = 44		N = 256		N = 123	
	N	w.%	N	w.%	N	w.%	N	w.%	N	w.%
Demographics										
Sex^d^										
Male	5	23.7	29	40.0	28	70.0	190	71.7	84	68.0
Female	20	76.3	44	60.0	16	30.0	66	28.3	39	32.0
Age^d^										
<17 years	0	–	0	–	1	0.0	6	1.9	3	0.0
17–64 years	1	2.6	59	78.6	35	88.9	198	77.4	97	79.2
65–79 years	4	15.8	8	14.3	6	11.1	43	15.1	18	16.7
80+ years	20	81.6	6	7.1	2	0.0	9	5.7	5	4.2
Marital status^d^										
Married	6	21.6	16	26.7	8	18.2	103	41.5	28	24.0
Unmarried	2	8.1	29	40.0	25	45.5	89	32.1	51	40.0
Divorced	0	–	20	26.7	7	27.3	50	18.9	34	28.0
Widowed	17	70.3	8	6.7	4	9.1	14	7.5	10	8.0

Situations that were applicable to the person according to the physician: e										
Medical conditions:										
Somatic disease^d^	17	60.5	17	26.7	14	40.0	38	17.0	19	16.0
Psychiatric disease	0	–	55	73.3	31	77.8	149	57.4	65	52.0
Dementia^d^	7	34.2	0	0.0	0	–	0	–	1	0.0
Accumulation of health problems related to old age^d^	14	63.2	4	6.7	1	0.0	6	5.6	3	4.0
Any of the above, of which	24	94.3	61	83.7	38	88.8	170	66.6	74	60.0
- Only psychiatric disease	0	–	41	57,1	23	44.4	129	48.8	54	44.0
- Other combinations	24	94.7	20	28.6	15	44.4	41	18.5	20	16.0
Psychosocial or existential problems^d^	1	2.6	18	26.7	19	50.0	88	33.3	39	32.0
Earlier requested physician assistance in dying suicide^d^	10	44.7	9	13.3	4	10.0	5	1.9	3	4.0

a Due to the weighting procedure the percentages that are reported cannot be derived from the absolute unweighted absolute numbers.

b VSED = Voluntary Stopping Eating and Drinking.

c MED = self-ingesting self-collected medication.

d The differences between the 5 groups are statistically significant (Fisher Freeman Halton Exact test; p < 0.001).

e More than one answer possible.

The majority of people who ended life by MED were female (60%), between 17 and 64 years old (79%), and were unmarried or divorced (67%) (see Table 2). According to the physician, a great majority (73%) suffered from psychiatric diseases, with more than half (57%) solely from psychiatric diseases. About a quarter (27%) suffered from psychosocial or existential problems. About one in every eight (13%) requested PAD under the Dutch PAD law.

People who ended life by MED were similar to people who ended life through other non-violent, violent or unknown methods. The majority of each of the four groups was between 17 and 64 years old (respectively 79%, 89%, 77% and 79%), and most were unmarried (respectively 40%, 46%, 32% and 40%) or divorced (respectively 27%, 27%, 19% and 28%). According to the physician many suffered solely from psychiatric diseases (respectively 57%, 44%, 48% and 44%), or had psychosocial or existential problems (respectively 27%, 50% 33% and 32%). Finally, the number of people who had requested for PAD under the Dutch PAD law was low (respectively 13%, 10%, 2% and 4%) (see Table 2).

People who ended life by VSED had the highest percentage of females, over 80 years old, widowed persons, an accumulation of health problems related to old age, somatic diseases, dementia, and requests for PAD and the lowest percentage of psychosocial or existential problems and psychiatric illnesses compared to all other methods of ending one's own life.

### 3.3 The context of a medical condition

To investigate the context of a medical condition in intentionally ending one's own life, the group of people who intentionally ended their own life and had a medical condition according to the physician (N = 367) was divided into two groups: people with solely psychiatric diseases (N = 120) and people with (a combination of) other diseases (N = 247) (see Table 3).

**Table 3:** Characteristics of people who ended their own life according to whether they had medical conditions (n = 521; absolute unweighted numbers and weighted percentages)^a^.

	Medical conditions				No medical conditions	

	Only psychiatric condition(s) n = 247		All other combinations n = 120		N = 154	
	n	w.%	n	w.%	n	w.%
Demographics						
Sex^b^						
Male	151	61.2	76	40.7	111	68.8
Female	96	38.8	44	59.3	43	31.3
Age^b^						
<17 years	4	2.0	0	–	6	3.1
17–64 years	220	88.0	52	20.3	118	75.0
65–79 years	23	10.0	34	22.0	22	12.5
80+ years	0	–	34	57.6	8	9.4
Marital status^b^						
Married	65	28.6	42	27.6	54	36.4
Unmarried	108	42.9	26	13.8	62	36.4
Divorced	67	26.5	19	8.6	25	15.2
Widowed	7	2.0	33	50.0	13	12.1

**Earlier requestedphysicianassistance in dying**^b^	3	4.1	17	27.6	11	6.3

a Due to the weighting procedure the percentages that are reported cannot be derived from the absolute unweighted absolute numbers.

b The differences between the 3 groups are statistically significant (Fisher Freeman Halton Exact test; sex p = 0.018; age p < 0.001; marital status p = 0.021; earlier request p = 0.010).

The majority of people who intentionally ended their own life and had (a combination of) medical conditions other than solely psychiatric diseases according to the physician were female (59%), 65 years or older (80%), and widowed (50%), and one in four (28%) requested PAD. They differed from people who intentionally ended their own life who solely had psychiatric diseases according to the physician, who were more often male (61% versus 41%), more often between 17 and 64 years old (88% versus 20%), less often over 80 years old (0% versus 58%), more often unmarried (43% versus 14%) or divorced (27% versus 9%), less often widowed (2% versus 50%), and had requested PAD less often (4% versus 28%) (See Table 3).

People who intentionally ended their own life and did not have a medical condition according to the physician (N = 154) were similar to people who intentionally ended their own life and had solely psychiatric diseases concerning gender, age, and requests for PAD. About two thirds (61–69%) were male, the great majority (75–88%) were between 17 and 64 years old, and about one in twenty requested PAD. Among the people without a medical condition, a small group of over 80 years old (9%) was present. This group of over 80 years old was not present within the group of people with solely psychiatric diseases (0%), but were well represented in people with combinations of medical conditions (58%).

## 4 Discussion

### 4.1 Summary

In the Netherlands, in 1.85% of all deaths in 2015 people ended their own life, of which 0.50% through VSED, 0.20% through MED, 0.13% through other non-violent methods and 0.70% through violent methods. The large majority of the people who ended life by VSED were over 80 years old, widowed, and female, and suffered from an accumulation of health problems related to old age, somatic problems, and dementia. Psychosocial and existential problems were not reported, and almost half had requested PAD. They differed from people who ended their own life through other methods, who were mostly under 65 years old, unmarried, suffering from psychiatric, psychosocial and existential problems, and had hardly requested PAD. People who ended their own life and had (a combination of) medical conditions other than solely psychiatric diseases were primarily over 65 years old, and one in four had requested PAD. They differed from the people without a medical condition or with solely psychiatric conditions, who were mostly under 65 years old, and had hardly requested for PAD.

### 4.2 Strengths and limitations

Major strengths of this study are the large nationwide sample, which is representative of all deaths in the Netherlands in 2015, the high response rate and few missing data. Also, there are only a few cases in which the physician's estimate there are ‘indications the patient has ended life him or herself’ does not coincide with the official death registry. This can be an affirmation of the reliability of the data. A limitation of this study is that the patient's perspective is lacking as the questionnaires are filled in by the attending physician. Related to this, while we know whether patients had a medical condition, it is not known to what extent these condition(s) contributed to the wish to end life. Perhaps other non-medical reasons (like loneliness) could have played a role, making the presence or absence of a medical condition less relevant. Finally, the attribution of cases to the group of violent or non-violent methods can be debated as we lacked information on the presence or absence of an intermediate phase of deep sleep. Determining the violence or non-violence of methods like intoxication and suffocation depends largely on the presence or absence of an intermediate phase of deep sleep (Chabot, 2007).

### 4.3 Incidence of suicide or intentionally ending one's own life

The estimate of the number of people ending their own life in the Netherlands in 2015 in our study is higher than the estimate of the number of suicides by Statistics Netherlands, namely 1.85% versus 1.3% of all annual deaths (Statistics Netherlands, 2020a). This difference can be explained by differences in interpreting which forms of dying are regarded as suicide or intentionally ending one's own life. For example, Statistics Netherlands does not include people who pass away by VSED, estimated at 0.50% of all annual deaths. In 99% of the cases VSED is registered as a natural death. There is debate whether VSED should be regarded as suicide (Den Hartogh, 2014; Jox et al., 2017; Van den Brink, 2015; Vink, 2014). It is also debated whether physician assisted suicide under the Dutch Termination of life on request and assisted suicide review procedures Act could be regarded as a suicide (Yuill, 2013), which is estimated at 0.1% of all annual deaths (Onwuteaka-Philipsen et al., 2017).

The estimation of the frequency of VSED and MED from our physician-based study is lower than these estimates from a population-based study (Chabot & Goedhart, 2009). This can be explained by the finding that physicians – who fill out the death certificates in the Netherlands – are not always aware of death wishes of their patient (Pasman, Willems, & Onwuteaka-Philipsen, 2013). A patient – especially at old age – who has ended life by MED might be incorrectly registered as a natural death. Also, anecdotal evidence exists where physicians are aware of the death wish and the patient having ended their own life, but register a natural death to prevent stigma for bereaved ones who otherwise will have to deal with the arrival and involvement of the police and the public prosecutor (Chabot, 2001, 2007; Vink, 2013). In this study, we found three cases of MED that did not have suicide as the registered cause of death.

### 4.4 Demographic and medical characteristics of people who ended life through VSED and MED

In line with others studies into MED most people who ended life by MED are under 65 years old and without a partner (Van der Heideet et al., 2012; Chabot, 2007; Chabot & Goedhart, 2009). These characteristics are different for people who ended life by MED *after* receiving counselling from counsellors working in cooperation with a right-to-die organisation. They were more often over 65 years old, more often had somatic diseases, an accumulation of problems related to old age and dementia, less often had psychiatric problems, and had more often requested PAD (Hagens, Pasman, & Onwuteaka-Philipsen, 2020). These characteristics were similar to those of people who intentionally ended life by VSED.

People who ended their own life by VSED differed from people who had chosen other methods than VSED. Amongst others, they were older than people ending life by MED. This might be explained by the fact VSED is not advised for people under 60 years without a life threatening disease (KNMG & V&VN, 2014). Compared to other studies into VSED (Van der Heideet et al., 2012, Bolt, Hagens, Willems, & Onwuteaka-Philipsen, 2015; Chabot & Goedhart, 2009), this study finds a greater percentage of people ending life by VSED that were over 80 years old (82% as to respectively 48–75% in the other studies), and females (78% as to respectively 51–62%). Furthermore, the absence of psychosocial or existential problems in the group of people who had ended life by VSED contrasts with other studies that find that existential problems play a major role in the patients’ motives to decide to hasten death by VSED (Bolt, Hagens, Willems, & Onwuteaka-Philipsen, 2015; Chabot, 2007; KNMG & V&VN, 2014). Perhaps the reporting physicians focus less on the psycho-existential suffering, and more on physical suffering as has been reported in other studies (Pasman, Rurup, Willems, & Onwuteaka-Philipsen, 2009; Fisher et al., 2009). This, however, does not explain why psycho-existential suffering has been found in other groups. Another explanation can be that these patients have not discussed their psycho-existential problems with the physician. Especially in relation to requests for PAD – which almost half of the people who ended life by VSED had requested – patients might be aware there is more chance that their request will be granted if they emphasize the somatic problems and problems related to old age instead of mentioning feelings of completed life or existential suffering (Onwuteaka-Philipsen et al., 2017; Chabot, 2019; Van Tol et al., 2008).

### 4.5 The context of a medical condition

Many people in this study who ended their own life suffered from a medical condition. These people potentially would have had access to PAD under the Dutch PAD law, that is if the request stemmed from their medical condition and under condition that all criteria of due care could be met (Termination of life on request and assisted suicide review procedures Act, 2001). Yet only a minority had requested PAD. Possibly people value their autonomy and self-determination and prefer to take their own responsibility and end their own life (Hagens, Onwuteaka-Philipsen, & Pasman, 2017). Other explanations are that requests for PAD might be impeded by a disturbed relationship with one's own physician, fear of provisional detention (especially for those patients with a psychiatric disease when physicians infer patients are ‘a risk to themselves’), or the conviction that PAD is not possible in their specific situation (Hagens, Onwuteaka-Philipsen, & Pasman, 2017). People who ended their own life in the absence of a medical condition or with solely psychiatric conditions rarely requested PAD under the Dutch PAD law. This could reflect awareness of patients that most physicians consider providing PAD to people with psychiatric problems or to people without a serious illness inconceivable (Bolt et al., 2012, Onwuteaka-Philipsen et al., 2017). This also illustrates that more factors than the presence of absence of a medical condition and whether this contributes to a wish to die may be relevant in whether people who want to end their own life wish to realize this via PAD.

The requirement of having a medical condition reflects the fact that PAD in the Netherlands is primarily embedded in the medical domain as it is presently understood in Dutch law. In this study, however, a minority of people who ended their own life did not have a medical condition. Their wishes to die could have originated from non-medical traumatic life events, like for example abuse, death or divorce of a partner or because they feel their life is completed (Rurup et al., 2011; Van Wijngaarden et al., 2015). This raises the question how to address the desire to die from people whose wish to end life is not rooted in a medical condition and therefore falls outside the medical framework of assistance in dying. Recently, a law for assistance in suicide for people over 70 years old that does not require a medical condition has been proposed (Dijkstra, 2020). This study, however, shows that of the people without a medical condition who ended their own life, relatively few were over 65 years old. These results are in line with two recent studies that found only small groups of older people with an active wish to die that did not stem from a medical condition (Hartog et al., 2020), or in absence of a medical condition (Kox, Pasman, Huisman, Benneker, & Onwuteaka-Philipsen, 2021). This seems to confirm the statement from the Committee Completed Life that the group of older people without an accumulation of problems of old age and without a medical condition who end their own life seems small (Schnabel et al., 2016), and questions the necessity for such a far reaching and controversial law. However, the Committee Completed Life gave several recommendations that could result in preventing people experiencing their life as completed. At the same time they recognized that in the current situation people do have the possibility to end one's own life by non-violent means, for example by VSED, and can receive non-punishable assistance with this (Schnabel et al., 2016).

### 4.6 Conclusion

Estimating the frequency of suicide or intentionally ending one's own life is influenced by definitions and information sources. Few people who had intentionally ended their own life requested PAD, especially those suffering from solely psychiatric diseases and those without a medical condition. Possible explanations may be the wish to take one's own responsibility, a disturbed relationship with one's own physician, the fear of provisional detention or an awareness of patients about the (in)conceivability of physicians to grant requests for PAD under the Dutch PAD law in certain situations. PAD in the Netherlands is embedded in the medical domain as it is presently understood in Dutch law. This raises the question how to address the desire to die from people whose wish to intentionally end their own life is not rooted in a medical condition and therefore fall outside the medical framework of assistance in dying.

## Ethics approval statement

According to Dutch policy (Personal Data Protection Act, 2015) the study did not require review by an ethics committee. Informed consent of the certifying physicians was assumed on return of the survey.

## Financial disclosure statement

This study was funded by the 10.13039/501100001826Netherlands Organisation for Health Research and Development (10.13039/501100001826ZonMw, project number 3400.8003). The funding source had no in volvement in the collection, analysis, and interpretation of data, in the writing of the articles and in the decision to submit it for publication.

## CRediT authorship contribution statement

**Martijn Hagens:** Conceptualization, Writing – original draft, Visualization. **H. Roeline W. Pasman:** Conceptualization, Writing – review & editing, Supervision. **Agnes van der Heide:** Conceptualization, Methodology, Project administration, Writing – review & editing, Funding acquisition. **Bregje D. Onwuteaka-Philipsen:** Conceptualization, Methodology, Validation, Formal analysis, Investigation, Data curation, Writing – review & editing, Visualization, Supervision, Project administration, Funding acquisition.

## Declaration of competing interest

The authors declare that they have no competing interests.
